# Use of Adult Stem Cells for Cartilage Tissue Engineering: Current Status and Future Developments

**DOI:** 10.1155/2015/438026

**Published:** 2015-07-09

**Authors:** Catherine Baugé, Karim Boumédiene

**Affiliations:** ^1^Normandie Université, 14032 Caen, France; ^2^UNICAEN, EA4652 MILPAT, 14032 Caen, France

## Abstract

Due to their low self-repair ability, cartilage defects that result from joint injury, aging, or osteoarthritis, are the most often irreversible and are a major cause of joint pain and chronic disability. So, in recent years, researchers and surgeons have been working hard to elaborate cartilage repair interventions for patients who suffer from cartilage damage. However, current methods do not perfectly restore hyaline cartilage and may lead to the apparition of fibro- or hypertrophic cartilage. In the next years, the development of new strategies using adult stem cells, in scaffolds, with supplementation of culture medium and/or culture in low oxygen tension should improve the quality of neoformed cartilage. Through these solutions, some of the latest technologies start to bring very promising results in repairing cartilage from traumatic injury or chondropathies. This review discusses the current knowledge about the use of adult stem cells in the context of cartilage tissue engineering and presents clinical trials in progress, as well as in the future, especially in the field of bioprinting stem cells.

## 1. Cartilage, a Tissue with Low Capacity of Repair

**Figure 1:**
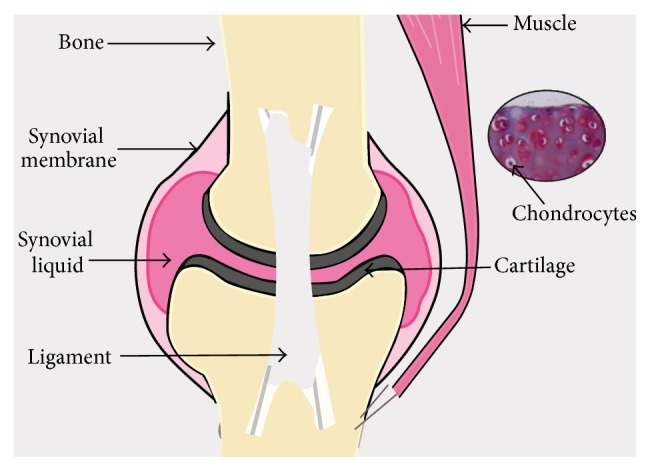
An overview of a typical joint structure. Hyaline cartilage, the most common type of cartilage, is located at the extremity of bones. It protects the subchondral bone acting as a lubricant and a shock absorber. It contains a single type of cells, called chondrocytes, maintained in an abundant matrix rich in collagen and proteoglycans.

Articular cartilage is a stable tissue that functions for decades to keep normal joint movement possible. It is a hyaline tissue with no blood, lymphatic or nerve supply. It contains a single type of cells, called chondrocytes (cell density: 1,500 to 2,000/mm^3^), maintained in an abundant connective tissue (Figure 1). This highly hydrated extracellular matrix (70–80%) is composed of collagen fibers, mainly type II collagen, and proteoglycan aggregates, mainly aggrecan, attached along a filament of hyaluronic acid. Collagens provide tensile strength, while proteoglycans are responsible for the compressive strength. The whole forms a viscoelastic structure well suited for both functions of cartilage: the absorption and distribution of forces and the sliding of the joint surfaces with a very low coefficient of friction. Thus, cartilage protects the subchondral bone acting as a lubricant and a shock absorber [1, 2]. Chondrocytes are responsible for the synthesis of this matrix. Indeed, these cells respond to growth factors, cytokines, or joint loading, by synthesizing extracellular matrix molecules. At opposite, chondrocytes can also express enzymes such as metalloproteases, favoring cartilage turnover and renewal.

During life, articular cartilage defects may happen and form areas of damaged or missing cartilage. These defects are often caused by acute trauma. Biochemical changes due to age may also stimulate the degradation of cartilage matrix and at term lead to chronic diseases such as osteoarthritis. These defects are the most often irreversible, since articular cartilage has very limited self-repair capability. This is partially due to its avascularity. With the lack of blood supply, a set of complex biochemical events that take place in order to repair the damage fails to occur. In addition, wound healing in hyaline cartilage is prevented by the dense extracellular matrix, which impairs the migration of chondrocytes [3–5]. However, a repair process may be initiated by undifferentiated mesenchymal stem cells (MSCs) from the bone marrow tissue of subchondral bone [6, 7]. Small full thickness defects can be repaired by formation of hyaline cartilage, whereas large osteochondral defects are only repaired by formation of scar tissue (fibrous tissue) or fibrocartilage. This fibrocartilage is a poorly organized tissue containing significant amounts of collagen type I. It exhibits inferior mechanical and biochemical characteristics compared to normal hyaline articular cartilage. The matrix of fibrocartilage breaks down with time and loading, leading to development of secondary OA in injured cartilage.

Consequently, when damaged, cartilage repair and regeneration is a major challenge. Currently, therapies such as microfracture, abrasion, drilling, and osteochondral grafting are applied and help to reduce pain to some degree. However, complications and injuries are huge and the results are largely unsatisfactory. In this context, regenerative cartilage medicine has been developing since 1987, with the first transplantation of autologous chondrocytes [8]. More recently, cell therapies based on adult stem cells have emerged.

## 2. Cartilage Tissue Engineering by Autologous Cell Implantation

**Figure 2:**
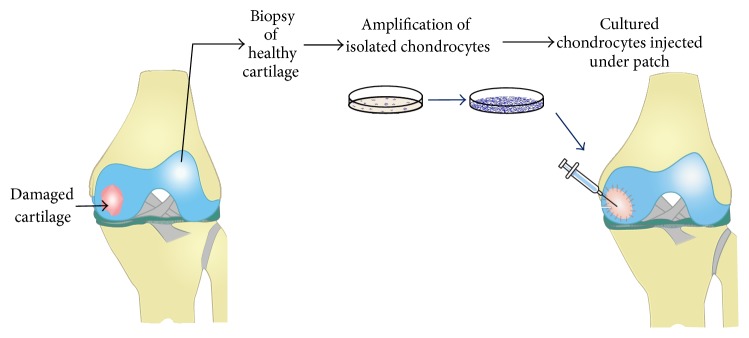
Schematic representation of autologous chondrocyte implantation (ACI) procedures. First, chondrocytes are extracted arthroscopically from the patient's healthy articular cartilage (nonload-bearing area of either the intercondylar notch or the superior ridge of the femoral condyles). Then, chondrocytes are expanded* in vitro* for approximately four to six weeks. Finally, once a sufficient number of cells have been obtained, the patient undergoes a second surgery where the cultured and amplified chondrocytes are applied to the damaged area. These transplanted cells grow in their new environment, forming new articular cartilage.

Cartilage is an attractive candidate for use in tissue-engineering therapies since this tissue is avascular and has a limited capacity for repair. With regard to chondrocyte implantation, an autologous strategy is preferred because of the risks associated with allogenic strategies, such as triggering an immunogenic response or transferring diseases. Autologous chondrocyte implantation (ACI) procedures take place in three stages (Figure 2). First, chondrocytes are extracted arthroscopically from the patient's healthy articular cartilage (nonload-bearing area of either the intercondylar notch or the superior ridge of the femoral condyles). Then, chondrocytes are expanded* in vitro* for approximately four to six weeks. Finally, once a sufficient number of cells have been obtained, the patient undergoes a second surgery where the cultured and amplified chondrocytes are applied to the damaged area. These transplanted cells grow in their new environment, forming new articular cartilage [9].

The use of autologous chondrocyte implantation may represent a promising technology for cartilage repair in orthopedic research. However, we and other investigators have established that, during monolayer expansion of chondrocytes* in vitro*, this cell population loses its phenotype, as illustrated by a switch in collagen production from type II (typical of hyaline cartilage) toward types I and III (typical of fibrocartilage) [10–12]. The result of these phenotype changes is the production of an extracellular matrix with inferior biomechanical properties. In addition, the limited capacity of the donor site to provide a large amount of chondrocytes, as well as donor site morbidity, is major obstacles for autologous chondrocytes. Therefore, use of stem cells, such as mesenchymal stem cells (MSCs), may be preferred. MSCs can be relatively easily harvested and the procedures using them are less invasive or destructive than articular cartilage harvesting procedures.

## 3. Sources of Mesenchymal Stem Cells for Cartilage Engineering

Mesenchymal stem cells (MSCs) have been heralded as the next major development in the repair and regeneration of cartilage. They are considered as an appropriate candidate owing to several specific characteristics (inherent chondrogenic property, easy availability, and cell homing potential). However, their use is still limited, with only 16% of reported cell therapy procedures for cartilage repair using MSCs, with the remaining chondrocytes [13].

### 3.1. Bone Marrow MSCs

MSCs, as nonhematopoietic cells, are originally derived from bone marrow tissue. In the 60s, scientists found that marrow cells are able to produce cartilage and bone-like tissue* in vivo* [14], but they were unable to determine the cells responsible for this property. Then, a fibroblastic population was isolated and described as the cellular equivalent of chondrogenic and osteogenic features of marrow tissue. Initially referred to as colony forming unit fibroblasts, these cells are now called mesenchymal stem cells (MSCs).

MSCs possess two important properties, long-term self-renewal ability and the capacity to differentiate along multiple cell lineages, such as bone, cartilage, and adipose cells. In spite of their limited numbers in bone marrow aspirate (the frequency of MSCs in the whole bone marrow of skeletally mature adults ranges from 1 in 50 000 to 1 in 100 000 cells, corresponding to a yield of a few hundred MSCs/milliliter of marrow), mesenchymal stem cells are easily expandable through standard culture techniques. Upon cultivation, they assume a spindly shaped morphology. MSC primary culture has been reported to be heterogeneous, containing multiple colonies with various differentiation capacities: nearly one-third of these colonies have osteogenic, adipogenic, and chondrogenic differentiation potentials, while the other two thirds exhibit either bipotent or unipotent capacity to differentiate into osteogenic/chondrogenic and adipogenic lineages, respectively [15].

### 3.2. Other Sources of MSCs

Besides bone marrow, multiple tissues have been reported to contain MSCs. These include adipose tissue, trabecular bone, periosteum, synovial membrane, and skeletal muscle, as well as teeth and umbilical cord [16]. Furthermore, some researchers have paid special attention to synovial membrane as a potent source of stem cells with good chondrogenic potential.

Unlike bone marrow MSCs, adipose MSCs can be isolated in large quantities with minimal morbidity and discomfort [17, 18]. The frequency of MSCs in adipose tissue is in the order of 1 in 100 cells, about 500-fold more than that found in bone marrow [19]. In view of these practical advantages, MSCs from adipose tissue could be considered an alternative option for bone marrow MSCs in cell-based cartilage regeneration strategies.

MSCs derived from synovial membranes also possess multilineage potential. These cells can be stimulated to undergo chondrogenesis* in vitro* with appropriate inducers. A study by Shirasawa et al. [20] showed that human synovial-derived cells have greater chondrogenic potential than bone marrow MSCs, adipose MSCs, and periosteal- or muscle-derived cells from the same patients. Furthermore, a follow-up study by the same authors indicated that synovial-derived MSCs produce consistently larger cartilage than bone marrow MSCs from the same patients [21].

In light of their availability, MSCs from skeletal muscle are also attractive sources of cells for use in cartilage tissue engineering because it is the largest organ in the body and only a minimally invasive procedure is required to harvest the tissue* via* muscle biopsy. Muscle has been extensively investigated as a potential source for isolation of pluripotent stem cells that can differentiate into various lineages, including myogenic, hematopoietic, and osteogenic [22, 23]. Concerning chondrogenic differentiation, it has been reported that the use of mice muscle-derived stem cells implantation to repair articular defects improved the healing of the defect, by inducing hyaline cartilage formation with an efficiency equivalent to chondrocyte transplantation [24–26].

Finally, another promising source of progenitor cells is umbilical cord blood (CB), even if it contains heterogeneous populations of stromal cells. Nevertheless, they were described as less mature than bone marrow MSCs and therefore open larger potential in regenerative medicine. Several subclinical or clinical trials are performed for now in the field of cartilage therapy [27]. However, classification of umbilical CB progenitor cells in subclasses is still uncompleted and further isolation of new markers will help in the characterization of the precise population that triggers chondrogenesis.

## 4. Differentiation towards Chondrogenesis

Repair or regeneration of cartilage depends on several parameters. When used, adult stem cells trigger chondrogenesis through a combination of events, including adaptation to a hypoxic milieu and activation of Sox proteins pathway that promote cells to produce their own cartilaginous matrix (type II collagen and aggrecan) [11]. Trophic factors are also of importance in this process. Among them, TGF beta members are widely used according to their well-stated role in the promotion of cartilage markers by MSC. However, it is likely that they act by a specific and sequential manner as described recently in a study that used a miRNA that regulates early chondrogenesis [28, 29]. Hence, the role of miRNAs emerges also as crucial in chondrogenesis and its elucidation will give more accurate information in the process of chondrogenesis. Finally, terminal differentiation is achieved by mechanical loading as well as hydrodynamic pressure, known to activate Sox-9 pathway and therefore chondrogenesis [30].

## 5. Culture Medium

The differentiation of adult stem cells into different cell types, especially to produce cartilage tissue, is reliant on the local microenvironment, growth factors, and extracellular matrix [31, 32].

Cell culture confers a highly controlled artificial environment on cells that can greatly modify their behavior. Classically, expansion protocols use a base medium, such as Dulbecco's modified eagle medium (DMEM), containing 10% fetal calf serum, maintained in a controlled 37°C atmosphere of 5% CO_2_ and ambient oxygen (~20%). Taking this to represent baseline conditions, numerous attempts have been made to modify the culture conditions in order to maintain a more stem-like nature of the resulting cells or favor chondrogenic differentiation.

A defined medium for* in vitro* chondrogenesis of MSCs was first reported, in 1998, by Johnstone et al., who used micromass culture with TGF-*β* and dexamethasone [32]. More recently, the necessity of cultured MSCs in medium containing dexamethasone to induce chondrogenic differentiation [33] and to add growth factors belonging to the TGF-*β* superfamily that constitute the earliest signals in chondrogenic condensation [34] was reported. Thus, medium is frequently supplemented with TGF-*β* superfamily members.

TGF-*β*1 is an important growth factor in tissue engineering for cartilage repair. It has been shown to promote chondrocyte proliferation and differentiation, both of which are important features of effective cartilage regeneration [35]. TGF-*β* is also known to be a potent inducer of stem cells chondrogenic differentiation and to favor the differentiation of MSCs to form ectopic cartilage* in vivo*. Supplementation with TGF-*β*1 could initiate and promote chondrogenesis of synovium-derived stem cell, but TGF-*β*1 alone was insufficient to fully differentiate these cells into chondrocytes. In muscle stem cells, our group also confirms the importance of TGF-*β*1 not only in enhancing the chondrogenesis, but also in maintaining the chondrogenic phenotype. In these stem cells, we showed that TGF-*β*1 enhances GAGs deposition, aggrecan, and type II collagen synthesis. Moreover, type I and type X collagen syntheses were inhibited, particularly in CD56−cells, suggesting that chondrocytes did not demonstrate significant hypertrophy. This data was further supported by the inhibition of nuclear proteins binding activity on Cbfa1 consensus sequence in TGF-*β*1-treated muscle stem cells, which could explain TGF-*β*1-induced downregulation of type I and type X collagens expressions [26]. TGF-*β*1 has also been shown to inhibit terminal differentiation of chondrocytes [36, 37].

However, findings concerning TGF-*β*1 effect on MSCs are still controversy and underline the fact that its effect on osteoblastic or chondrogenic differentiation of MSCs depends on culture conditions and doses used.* In vitro* experiments demonstrated that its use may lead to hypertrophic differentiation, with subsequent formation of an inadequate tissue instead of hyaline cartilage [38, 39]. In addition, a high dose of TGF-*β*1* via* intra-articular injection is known to induce chemotaxis and activation of inflammatory cells, resulting in characteristic cartilage defects such as fibrosis and osteophyte formation [35]. Therefore, it is evident that TGF-*β*1 should be administered in a controlled manner to minimize adverse effects.

Several other growth factors are known to influence the anabolic and catabolic processes of chondrocytes. Therefore, a number of these growth factors have been used in cartilage tissue engineering studies* in vitro* to promote the chondrogenic phenotype, to stimulate extracellular matrix production, and to promote chondrogenesis of MSCs. Among others, TGF*β*-3 stimulates the synthesis of proteoglycans and collagens matrix components [40] and is necessary for different steps during chondrogenesis. It probably acts by inducing the expression of Sry-related high-mobility-group box-9 (Sox-9) [41], which in turn regulates the expression of aggrecan and collagen type II, type IX, and type XI during chondrocyte differentiation.

Furthermore, research has indicated that addition of bone morphogenetic proteins (BMPs) also enhances chondrogenesis and can be used for the development of cartilage engineering strategy. BMPs have multiple important roles during skeletal formation [35]. BMP-2, -5, and -6 maintain and promote later stages of chondrocyte differentiation rather than initiation of maturation [42], while BMP-7 (also referred to as osteogenic protein 1, OP-1) promoted chondrocyte proliferation and inhibited terminal differentiation [43]. BMP-2 also regulates the chondrogenic differentiation and the maturation of mesenchymal progenitors and stimulates the synthesis of chondrocyte matrix components, [44]. For this reason, BMP-2 has been proposed as a tool for cartilage repair and as an inducer of chondrogenesis.

In cultures of human mesenchymal stem cells, BMP-2 as well as BMP-9 increases the synthesis of cartilage-specific proteins [35]. Comparing the ability of BMP-2, BMP-4, and BMP-6 to promote the differentiation of mesenchymal stem cells from bone marrow toward chondrocyte showed that BMP-2 appears to be the most effective. However, under BMP-2, mesenchymal stem cells can possibly continue their differentiation to hypertrophy and osteogenesis, characterized by type X collagen and Runx2 expression. Finding a way to reduce this adverse effect is subsequently essential to the development of an efficient strategy of tissue engineering for cartilage repair. Some researchers propose to cotreat cells with TGF-*β* and BMP-2. Pretreatment with TGF-*β* could prevent fully differentiation of MSCs into osteoblasts [45]. Although BMP-2 induces osteogenic and chondrogenic phenotypes in adipose-derived stem cells, TGF-*β*1 can inhibit BMP-2-induced differentiation of the osteogenic lineage, and combined growth factor treatment shows a synergistic effect on the expression of cartilage-specific genes and elevated release of cartilage-specific ECM proteins [46].

GDF-5 (growth differentiation factor-5) also known as BMP-14 or cartilage-derived morphogenetic protein (CDMP-1) shows also some capacity to stimulate cartilage matrix synthesis. It induces the differentiation of mesenchymal stem cells into chondrocytes and promoted increased accumulation of GAG and type II collagen during pellet culture [47].

Other cytokines, such as insulin-like growth factor (IGF), or parathyroid hormone related peptide (PTHrP) had been tried for better differentiation of stem cells, but it is still difficult to obtain “*in vitro*” MSC-based cartilage formation comparative to native cartilage tissue [32].

## 6. Biomaterials and Scaffolds

As evidenced by previous paragraph, growth factors are essential to induce chondrogenic differentiation of adult stem cells. However, to promote/maintain cartilage differentiation/phenotype in culture, another critical requirement is to provide a 3D microenvironment. Indeed, research has demonstrated that MSCs hardly differentiate into cartilage cell lineage in a 2D culture system.

The initial technique for chondrogenic differentiation of MSCs was the micromass culture system. The cells were placed in a tube and centrifuged into a condensed aggregate. To date, this technique is still widely used to evaluate chondrogenic potential of MSCs* in vitro*. MSCs cultured in micromass increase expression of type II collagen, a marker of chondrocyte phenotype, but they also increase the expression of hypertrophic marker, such as type X collagen [32].

For applications of cartilage tissue replacement, most investigators preferred transplantation of cells combined with scaffold. So, a huge expansion in biomaterial technologies and scaffolds took place to create functional tissue replacement to treat cartilage defects or osteoarthritis. Numerous biomaterials and scaffolds are being developed, influenced by the knowledge of the anatomical and structural complexity of articular cartilage. In addition to being biocompatible and accommodating cell adhesion, proliferation, and matrix synthesis, an ideal biomaterial scaffold for cartilage regeneration should be bioactive, biomimetic, biodegradable, and bioresponsive, providing signaling with spatiotemporal control and response that is selective to defined stimuli.

Natural and synthetic scaffolds have been developed. Natural materials, such as collagen, hyaluronic acid, or alginate, have advantage to be biodegradable, easily available, and bioactive. For their part, synthetic materials, such as polyethylene glycol (PEG), polyglycolic acid (PGA), polymethyl methacrylate (PMMA), or polylactide-co-glycolide (PLGA), are inert and have a long shelf-life. Their physical proprieties can also be easily changed (porosity, degradation time…) [9].

## 7. Clinical Trials

Many clinical trials have been registered at https://www.clinicaltrial.gov/ regarding application of stem cells for regenerating cartilage. About 40 studies (phase 1 to 3) are in progress or are completed worldwide (Table 1). Most of them aim to repair cartilage defects or treat degenerative damage, in knee, ankle, or hip, due to osteoarthritis. One clinical trial aims to implant a partial larynx.

**Figure 3:**
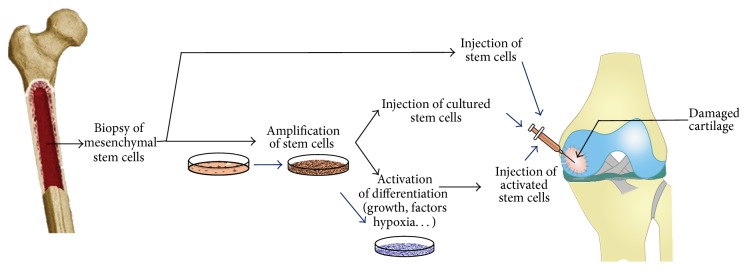
Schematic representation of cell therapy for cartilage based on stem cell implantation. Mesenchymal stem cells (MSCs) are isolated from different sources: bone marrow, fat tissues, or umbilical cord. Synovial-derived cells or MSCs derived from skeletal muscle can also be used. Then, these cells are either injected into damaged zone of cartilage or amplified* in vitro*. After amplification, stem cells are injected into damaged cartilage directly or after a stage of activation toward chondrogenesis by addition of growth factors, cultured in hypoxia and in an adapted scaffold.

Different strategies are tested (Figure 3). The simplest and most frequent one consists of intra-articular injection of mesenchymal stem cells, either directly after collection (i.e., fresh non cultured and expanded cells), or after amplification and culture during 2–4 weeks. Another strategy aims to mix stem cells with a scaffold for implantation. For this, different biomaterials are testing collagen hydroxylapatite scaffold, collagen I scaffold, or decellularized human donor scaffolds. Three sources of stem cells are used: bone marrow-derived mesenchymal stem cells, adipose-derived stem cells, and umbilical cord blood-derived mesenchymal stem cells.

Some preliminary results have been published and are promising. A 31-year-old male patient having received MSC/collagen gel into cartilage defects in his knee returned to a normal life seven months after implantation and the formation of hyaline cartilage in the histological sections has been observed [48]. Similarly, Wakitani et al. transplanted autologous MSCs combined with collagen gel into five patients with full thickness articular cartilage defects in their patellofemoral joints and observed significant improvements in patients' pain and walking ability six months after transplantation. However, one year after transplantation, histological examination of the repair tissue from one patient revealed that the defect was repaired by fibrocartilaginous tissue [49, 50]. The same group made also some attempts to treat osteoarthritic joints. In these clinical trials, adherent cells from bone marrow aspirates were embedded in collagen gel and transplanted into articular cartilage defects in the medial femoral condyle of 12 patients, while the other 12 subjects served as cell-free controls. Outcomes indicated that although clinical improvement was not significantly different, the treatment group showed a better arthroscopic and histological grading score [51]. In the above-mentioned study, MSCs were introduced through an invasive approach (surgery) into the defective area. Some authors have attempted to introduce the cells by injection. Using this approach, Centeno et al. [52] applied culture expanded autologous MSCs and transplanted the cells through an intra-articular injection into the knee of a 46-year-old OA patient. They reported that 90% of the patient's pain was reduced two years after injection. Furthermore, Davatchi et al. [53], Emadedin et al. [54], and Orozco et al. [55, 56] used this strategy to introduce the cells into knee joints of OA patients and reported the strategy as an encouraging method.

## 8. Pitfalls and Future Developments

In spite of the above-mentioned potential, there are some pitfalls associated with MSC application for articular cartilage regeneration. One is the qualities and mechanic properties of neoformed cartilage, and the second is the fabrication of anatomically relevant 3D engineered tissue and its integration into surrounding native joint tissues.

### 8.1. Improving Quality of Cartilage Implant

Quality of cartilage obtained after implantation of stem cells in joints is a major issue. Indeed, it has been reported that the thickness of the regenerated cartilage by MSCs transplanted into cartilage defects is too thin to resemble mature cartilage [57], and histological examination of the repair tissue from patients revealed that the defect was repaired by fibrocartilaginous tissue. A fear is also the risk of ossification of cartilage tissue [58–61]. Another major limitation for clinical applications is the presence of serum and growth factors in chondrogenic medium to induce chondrocyte phenotype, what raises cost of protocols and causes important ethic and regulation problems.

A major improvement of current methods to differentiate stem cells into chondrocytes will be developing culture conditions which will require neither serum nor growth factors. Several investigators, including us, have exploited low oxygen tension as a strategy for differentiated stem cells into chondrocytes. These studies have shown that hypoxia (1–3% O_2_) enhances COL2A1 and aggrecan expression and reduces expression of collagen types I and X [11]. Interestingly, 3D culture (alginate beads) associated with hypoxic environment is sufficient to differentiate human bone marrow-derived mesenchymal stem cells into chondrocytes, without addition of any growth factor [11].

Furthermore, gene therapy approaches could become a promising strategy for efficient promotion of regeneration in cartilage defects. MSC-based gene therapy offers some advantages for articular cartilage repair. Using this approach, specific genes could be overexpressed in MSCs before transplantation of these modified cells into articular cartilage defect. This in turn could enhance the structural features of the repair tissue formed at the defect site. Furthermore, MSC-based gene therapy is an applicable approach to deliver genes with complementary mechanisms of action (i.e., chondrogenic and proliferative factors) into a cartilage defect. In many studies, MSC-mediated gene delivery has been applied for cartilage repair using a variety of chondrogenic growth factors, such as IGF-1, TGF-*β*1 or BMP-2 [62–65], BMP-4 [25], and growth differentiation factor 5 [66].

Another way to improve cartilage engineering is to use other sources of stem cells. A recent evolution consists of studying potential of induced pluripotent stem cells (iPSCs) to differentiate in chondrocytes. iPSCs have pluripotency and self-renewal similar to embryonic stem cells (ESCs) but are not associated with the ethical issues related to sacrificing embryos for the generation of ESCs. The iPSCs were first generated, in 2006, by transducing mouse fibroblasts with four factors (c-Myc, Klf4, Oct3/4, and Sox-2), called reprogramming factors [65]. The efficiency of iPSC generation has been greatly improved since its initial discovery. Blood cells can also be converted to iPSCs [67], although the efficiency is lower than that associated with generating iPSCs from fibroblasts. When iPSCs are implanted into immunodeficient mice, teratomas are formed. Interestingly, hyaline cartilage is contained in these teratomas, confirming that iPSCs have the ability to differentiate into chondrocytes. Various approaches have been developed to induce the differentiation of iPSCs toward chondrocytes [68]. As a whole, the reports on these methods showed that the resultant cells expressed chondrocytic markers but rarely showed that the resultant cells could actually generate scaffold-free hyaline cartilage. It remains to be proven that chondrocytes differentiated from human iPSCs can definitely generate cartilage with the same characteristics as native tissue. In addition, the use of iPSCs is associated with a potential risk of tumor formation. However, several studies have implanted human iPSC-derived chondrocytes into immunosuppressed rats [69]. Cartilage was formed in the defects created in the articular cartilage of these rats, without any teratoma or tumor formation, suggesting that iPSC-derived chondrocytes are a promising source of cells for transplantation. The efficacy and safety of such transplantation remain to be investigated in more immunodeficient animals and larger animal models which would allow for a more accurate assessment of the repair capacity of the cells.

### 8.2. Improving Anatomy and Zonal Organization of Neoformed Cartilage

Another challenge in cartilage engineering approaches is the generation of cell-seeded implants with structures that mimic native tissue, in both anatomic geometries and cellular distributions [70]. The ideal implanted tissue should integrate with existing native cartilage and be able to repair lesions of different sizes and thicknesses. Existing processes are incapable of easily creating cartilage responding to these criteria, that is, with the required spatial heterogeneities and accurate anatomical geometries. Three-dimensional bioprinting should solve this difficulty in the next years. This technology consists in delivering living cells in suspension or with a gel as an ink, in layer-by-layer process. It should allow bringing cells with appropriate matrix material in a defined and organized manner, at the targeted location, in adequate numbers and within the right environment. One of the most critical challenges for use of 3D printing for tissue engineering is the integration of a vascular network, without which the engineered 3D tissue or organ cannot receive sufficient nutrients or gas for its regeneration [71]. However, cartilage has a relatively simple structure with no vessels or nerves, making cartilage a suitable tissue for 3D bioprinting.

Inkjet printing [72, 73], laser-based direct writing of cells [74–76], and extrusion-based cell-laden hydrogel deposition [77–79] are the most widely used technologies in development for tissue reconstruction. With these technologies, cells, scaffolds, and growth factors can be precisely deposited to the desired two-dimensional (2D) and three-dimensional (3D) locations rapidly. The bioprinting approach often utilizes naturally derived hydrogels, as inks to construct tissues due to their superior biocompatibility and low toxicity on the cells. For instance, collagen scaffold can be printed at 4°C and physically crosslinked at elevated temperature [80]. Alginate [72] and fibrin [81, 82] scaffolds can also be generated and used to print cells. For example, cartilage progenitor cells encapsulated in sodium alginate and printing through a pressure-assisted robotic bioprinting system are able to survive after bioprinting and to undergo differentiation with high-level cartilage-associated gene expression. However, some cells died probably due to mechanical stimulation during bioprinting [83]. These natural printed scaffolds have limited mechanical properties because of their nature, and the crucial phenomenon of integration with surrounding native tissues directly may be difficult. Therefore, developing of printable biomaterials, capable of simultaneous polymerization during printing with mechanical properties matched to native tissue, is critical for cartilage engineering. Synthetic hydrogels may be adapted. Thus, formulated from poly(ethylene glycol) (PEG) macromers are able to maintain chondrocyte viability and induce ECM deposition containing proteoglycans and type II collagen [84, 85]. Furthermore, polyethylene glycol dimethacrylate (PEGDMA) with human chondrocytes was also used to repair defect in osteochondral plugs in layer-by-layer assembly utilizing a 3D thermal inkjet-based bioprinting/biopolymerization method. Viability of printed cells was better in simultaneous polymerization than polymerized after printing. Interestingly, the printed PEG gel was firmly bound to the native tissue [86]. Despite its ability to control the scaffold porosity, the synthetic scaffold may not provide the proper affinitive environment to the cells due to the polymer hydrophobicity properties. In this context, hybrid scaffolds using both synthetic polymers and hydrogel materials may also provide a favorable environment within the hydrogel for the cells to grow and can possess more adequate mechanical properties permitting implants to bear loads [87].

## 9. Conclusion

Although initially considered as a tissue with a simple structure, reproducing the finely balanced structural interactions of cartilage has proven to be difficult. First clinical trials of cartilage regeneration using autologous chondrocyte implantation chondrocyte show major limits. In this context, adult stem cells, especially MSCs, appear as more appropriate cell candidates for regenerating incurable defects of articular cartilage due to the following characteristics: inherent chondrogenic property, easy availability, cell homing potential, and immunomodulatory function. However, special attention must be given to improve the quality of repair tissue formed following stem cells transplantation into the cartilage defect. Efficient protocols and optimal biomaterials must be developed to prevent hypertrophy of chondrocytes produced by MSC differentiation and to favour integration of implant into surrounding joint tissue. In addition, bioprinting technology, which has advantage to deliver cells, growth factors, and biomaterial scaffold precisely to the desired 3D position, may be the ultimate solution to engineer cartilage tissue.

## Figures and Tables

**Table 1:** List of clinical trials referenced in https://clinicaltrials.gov/ for the terms “stem cells and cartilage.”

Title	Country	Date	Status	Phase	Conditions	Intervention	Age of patients	Estimated enrollment	Results
Treatment of Knee Osteoarthritis with Autologous Mesenchymal Stem Cells	Spain	2010 to 2014	Completed with results	1 and 2	Osteoarthritis, knee; knee degenerative disease; knee Osteoarthritis	Autologous bone marrow mesenchymal stem cells (MSV) by articular injection	18–76	12	MSC therapy may be a valid alternative treatment for chronic knee osteoarthritis. The intervention is simple, does not require hospitalization or surgery, provides pain relief, and significantly improves cartilage quality. Orozco et al., 2013 [56]; Orozco et al., 2014 [55]
Treatment of Knee Osteoarthritis with Allogenic Mesenchymal Stem Cells	Spain	2012 to 2014	Completed with results	1 and 2	Osteoarthritis, knee; arthritis of knee; knee osteoarthritis	Allogenic mesenchymal stromal cells injection (compared to hyaluronic acid injection)	18–75	30	
Study to Compare the Efficacy and Safety of Cartistem and Microfracture in Patients with Knee Articular Cartilage Injury or Defect	Korea	2009 to 2011	Completed	3	Cartilage injury; osteoarthritis	Cartistem (allogeneic-unrelated, umbilical cord blood-derived mesenchymal stem cells, *ex vivo*, combined with sodium hyaluronate) (compared to microfracture treatment)	18 and older	104	
Transplantation of Bone Marrow-Derived Mesenchymal Stem Cells in Affected Knee Osteoarthritis by Rheumatoid Arthritis	Iran	2009 to 2011	Completed	2 and 3	Rheumatoid arthritis	Bone marrow mesenchymal cell transplantation; (compared to placebo)	18–65	60	
Articular Cartilage Resurfacing With Mesenchymal Stem Cells In Osteoarthritis Of Knee Joint	Iran	2009 to 2010	Completed	2	Osteoarthritis	Mesenchymal stem cells injection	18–65	6	
The Effects of Intra-articular Injection of Mesenchymal Stem Cells in Knee Joint Osteoarthritis	Iran	2010 to 2012	Completed	2	Osteoarthritis	Bone marrow mesenchymal stem cell (compared to placebo)	18–65	40	
Adult Stem Cell Therapy for Repairing Articular Cartilage in Gonarthrosis	Spain	2010 to 2013	Completed	1 and 2	Osteoarthritis, knee; knee Injuries; joint diseases; rheumatic diseases; cartilage diseases	Autologous mesenchymal stem cells (MSCs) knee implantation after “*ex vivo*” expansion	18–65	15	
Autologous Transplantation of Mesenchymal Stem Cells (MSCs) and Scaffold in Full-thickness Articular Cartilage	Iran	2008 to 2010	Completed	1	knee osteoarthritis	Bone marrow-derived mesenchymal stem cells mixed with collagen I scaffold	45–60	6	
Side Effects of Autologous Mesenchymal Stem Cell Transplantation in Ankle Joint Osteoarthritis	Iran	2010 to 2011	Completed	1	Osteoarthritis	Bone marrow-derived mesenchymal stem cell	18–65	6	
Mesenchymal Stem Cell Transplantation in Osteoarthritis of Hip Joint	Iran	2010 to 2011	Completed	1	Hip osteoarthritis	Bone marrow mesenchymal stem cell injection	18–65	6	
“One-step” Bone Marrow Mononuclear Cell Transplantation in Talar Osteochondral Lesions	Italy	2013 to 2016	Recruiting	3	Osteochondritis	Bone marrow-derived cells transplantation on collagen scaffold	15–50	140	
Mesenchymal Stem Cells in Knee Cartilage Injuries	Jordan	2014 to 2015	Recruiting	2	Articular cartilage disorder of knee; osteoarthritis, knee	Autologous mesenchymal stem cells	40–68	16	
Evaluation of Safety and Exploratory Efficacy of CARTISTEM, a Cell Therapy Product for Articular Cartilage Defects	USA and Korea	2012 to 2015	Recruiting	1, 2 and 3	Osteoarthritis, degenerative, degenerative injury, traumatic injury, knee	Cartistem (allogeneic-unrelated, umbilical cord blood-derived mesenchymal stem cells, *ex vivo*, combined with sodium hyaluronate)	18 and older	12	
Autologous Bone Marrow Mesenchymal Stem Cells Transplantation for Articular Cartilage Defects Repair	Brazil	2012 to 2014	Recruiting	1 and 2	Osteoarthritis	Bone marrow-derived mesenchymal stem cells	25–65	10	
Human Autologous MSCs for the Treatment of Mid to Late Stage Knee OA	Canada	2015 to 2021	Recruiting	1 and 2	Osteoarthritis of knee	Mesenchymal stem cells	40–65	12	
Mesenchymal Stem Cells Enhanced With PRP Versus PRP In OA Knee	India	2013 to 2014	Recruiting	1 and 2	Osteoarthritis, knee	Mesenchymal stem cell suspension	40–75	24	
Treatment of Osteoarthritis by Intra-articular Injection of Bone Marrow Mesenchymal Stem Cells With Platelet Rich Plasma	Spain	2014 to 2017	Recruiting	1 and 2	Knee osteoarthritis	Bone marrow mesenchymal stem cells (compared to Platelet Rich plasma (PRGF))	40–80	38	
Mesenchymal Stem Cells as a Treatment for Oral Complications of Graft-versus-host Disease	Sweden	2014 to 2017	Recruiting	1 and 2	Graft -versus-host-disease	Mesenchymal stromal cells	18–80	12	
Use of Autologous Bone Marrow Aspirate Concentrate in Painful Knee Osteoarthritis	USA	2013 to 2014	Recruiting	1	Bilateral primary osteoarthritis of knee	Autologous bone marrow aspirate	18 and older	25	
Bone Marrow Stromal Cells for Inflammatory Bowel Diseases	USA	2013 to 2019	Recruiting	1	Inflammatory bowel disease	Bone marrow stromal cell (BMSC) infusion	18–65	100	
Microfracture Versus Adipose Derived Stem Cells for the Treatment of Articular Cartilage Defects	USA	2014 to 2019	Recruiting		Degenerative lesion of articular cartilage of knee	adipose derived stem cells (compared to microfracture)	18–40	40	
Safety and Efficacy of Autologous Bone Marrow Stem Cells for Treating Osteoarthritis	India	2010 to 2012	Enrolling by invitation	1 and 2	Osteoarthritis	Autologous bone marrow stem cells	30–70	10	
Effectiveness and Safety of Autologous ADRC for Treatment of Degenerative Damage of Knee Articular Cartilage	Russian	2015	Enrolling by invitation	1 and 2	Osteoarthritis	Autologous bone marrow stem cells	20–85	12	
Follow-Up Study of CARTISTEM Versus Microfracture for the Treatment of Knee Articular Cartilage Injury or Defect	Korea	2012 to 2015	Active, not recruiting	3	Degenerative osteoarthritis; defect of articular cartilage	Cartistem (allogeneic-unrelated, umbilical cord blood-derived mesenchymal stem cells, *ex vivo*, combined with sodium hyaluronate) (compared to microfracture procedure)	18 and older	103	
IMPACT: Safety and Feasibility of a Single-stage Procedure for Focal Cartilage Lesions of the Knee.	Netherland	2013 to 2015	Active, not recruiting	1 and 2	Foreign-body reaction; inflammation; effusion (L) knee; knee pain swelling	Mesenchymal stromal cells mixed with fibrin cell carrier	18–45	35	
Treatment of Knee Osteoarthritis by Intra-articular Injection of Bone Marrow Mesenchymal Stem Cells	Spain	2012 to 2015	Active, not recruiting	1 and 2	Osteoarthritis	Bone marrow mesenchymal stem cells	50–80	30	
Autologous Adipose Stem Cells and Platelet Rich Plasma Therapy for Patients With Knee Osteoarthritis	Vietnam	2013 to 2015	Active, not recruiting	1 and 2	Knee osteoarthritis	Autologous adipose tissue stromal vascular fraction and platelet rich plasma	18 and older	32	
Mesenchymal Stem Cells in a Clinical Trial to Heal Articular Cartilage Defects	Norway	2009 to 2018	Active, not recruiting	1	Defect of articular cartilage	Mesenchymal stem cells or chondrocytes under a commercial available membrane	18–50	50	
Human Umbilical Cord Mesenchymal Stem Cell Transplantation in Articular Cartilage Defect	China	2014 to 2016	Not yet recruiting	1	Cartilage diseases; osteoarthritis	Human umbilical cord mesenchymal stem cells	18–75	20	
Clinical Trial of Stem Cell Based Tissue Engineered Laryngeal Implants	United Kingdom	2015 to 2018	Not yet recruiting	1 and 2	Disorder of upper respiratory system; laryngostenosis; tracheal stenosis	Stem cell based tissue engineered partial laryngeal implants (autologous-derived cells and decellularized human donor scaffolds)	18 and older	10	
The Use of Autologous Bone Marrow Mesenchymal Stem Cells in the Treatment of Articular Cartilage Defects	Egypt	2006 to 2014	Unknown	2 and 3	Degenerative arthritis; chondral defects; osteochondral defects	Bone marrow mesenchymal stem cell implantation (after culture expansion in pellet)	15–55	25	
Intra-Articular Autologous Bone Marrow Mesenchymal Stem Cells Transplantation to Treat Mild to Moderate Osteoarthritis	Malaysia	2011 to 2014	Unknown	2	Osteoarthritis	Autologous bone marrow-derived mesenchymal stem cells (compared to Hyaluronic Acid)	18–70	50	
Safety and Efficacy Study of Umbilical Cord-Derived Mesenchymal Stem Cells for Rheumatoid Arthritis	China	2013 to 2014	Unknown	1 and 2	Rheumatoid arthritis	Umbilical cord-derived mesenchymal stem cells (UC-MSCs)/rheumatoid arthritis with disease-modifying drugs (DMARDs)/: UC-MSC + DMARDS	18–70	200	
Autologous Mesenchymal Stem Cells versus Chondrocytes for the Repair of Chondral Knee Defects	Spain	2011 to 2012	Unknown	1 and 2	Articular cartilage lesion of the femoral condyle	Mesenchymal stem cells derived from adipose tissue	18–55	30	
Transplantation of Bone Marrow Stem Cells Stimulated by Proteins Scaffold to Heal Defects Articular Cartilage of the Knee	France	2010 to 2014	Unknown	0	Osteoarthritis; knee osteoarthritis; osteochondritis dissecans; osteonecrosis	Transplantation of nonculture expanded autologous bone marrow stem cells stimulated with a protein matrix and mixed in a collagen hydroxyapatite scaffold	30–75	50	
Peripheral Blood-derived Stem Cell Trial on Damaged Knee Cartilage	Malaysia	2009 to 2012	Unknown		Damaged articular cartilage	Peripheral blood-derived stem cell and hyaluronic acid	18–50	50	
